# Comparison of the collagen haemostat Sangustop^® ^versus a carrier-bound fibrin sealant during liver resection; ESSCALIVER-Study

**DOI:** 10.1186/1745-6215-11-109

**Published:** 2010-11-19

**Authors:** Christian Moench, Wolf O Bechstein, Valentin Hermanutz, Godehard Hoexter, Hanns-Peter Knaebel

**Affiliations:** 1 Department of General Surgery, Johann-Wolfgang Goethe-University, Theodor- Stern- Kai 7, 60590 Frankfurt, Germany; 2 Aesculap AG, Department of Clinical Science, Am Aesculap Platz, 78532 Tuttlingen, Germany; 3 Dr. M. Koehler GmbH, Pharma Biometrie Consulting, Hornusstr. 16, 79108 Freiburg, Germany

## Abstract

**Background:**

Haemostasis in liver surgery remains a challenge despite improved resection techniques. Oozing from blood vessels too small to be ligated necessitate a treatment with haemostats in order to prevent complications attributed to bleeding. There is good evidence from randomised trials for the efficacy of fibrin sealants, on their own or in combination with a carrier material. A new haemostatic device is Sangustop^®^. It is a collagen based material without any coagulation factors. Pre-clinical data for Sangustop^® ^showed superior haemostatic effect. This present study aims to show that in the clinical situation Sangustop^® ^is not inferior to a carrier-bound fibrin sealant (Tachosil^®^) as a haemostatic treatment in hepatic resection.

**Methods/Design:**

This is a multi-centre, patient-blinded, intra-operatively randomised controlled trial. A total of 126 patients planned for an elective liver resection will be enrolled in eight surgical centres. The primary objective of this study is to show the non-inferiority of Sangustop^® ^versus a carrier-bound fibrin sealant (Tachosil^®^) in achieving haemostasis after hepatic resection. The surgical intervention is standardised with regard to devices and techniques used for resection and primary haemostasis. Patients will be followed-up for three months for complications and adverse events.

**Discussion:**

This randomised controlled trial (ESSCALIVER) aims to compare the new collagen haemostat Sangustop^® ^with a carrier-bound fibrin sealant which can be seen as a "gold standard" in hepatic and other visceral organ surgery. If non-inferiority is shown other criteria than the haemostatic efficacy (e.g. costs, adverse events rate) may be considered for the choice of the most appropriate treatment.

**Trial Registration:**

NCT00918619

## Background

All surgical procedures inevitably lead to bleeding. Haemostasis - the control of bleeding - aims at reducing the amount of blood loss and the need for transfusion as well as preventing re-bleeding, haematoma formation with subsequent morbidities, and the need for intervention or repeat surgery. During liver resection the control of bleeding is a major concern. The liver is predisposed to a diffuse bleeding because of its extreme vascularity, particularly because of the hepatic sinusoidal structure, which does not have smooth muscles capable of contraction to induce vasoconstriction.

Surgical techniques and devices to facilitate haemostasis have been developed in the last decades and have minimised operative risks of liver resection. Nevertheless, a parenchymal transsection of the liver tissue is always associated with some degree of bleeding due to the division of small blood vessels which can not be isolated and ligated.

In order to achieve control over that parenchymatic diffuse bleeding from the resection surface and to prevent intraperitoneal complications attributed to bleeding various locally applicable agents (haemostats) are in use. These haemostats include bone wax, gelatine, collagen, oxidized regenerated cellulose, fibrin sealant glues, and synthetic glues [1]. Some evidence from randomized controlled trials (RCT) exists regarding the use of fibrin sealants on their own or combined with a collagen fleece [2,3].

A composite product with well documented efficacy is Tachosil^® ^[Nycomed, Linz, Austria]. It consists of a collagen fleece carrying the fibrin glue components human fibrinogen and human thrombin. It was shown in a RCT to be superior in obtaining intraoperative haemostasis over argon beamer in liver resection [4]. The time to haemostasis was significantly reduced. Also in kidney tumour resection a randomized study showed superiority over standard suturing [5].

A new haemostat product is Sangustop^® ^[Aesculap, Tuttlingen, Germany]. It is indicated for local haemostasis of capillary bleeding and bleeding of parenchymal organs. Sangustop^® ^is composed of native absorbable collagen fibrils without any blood serum products or any pharmaceutical activity. The felt structure being rich in surface gives a framework for the adhesion of blood platelets, thus provides an additional impetus to clotting. Pre-clinical data showed very good haemostatic activity, superior to sealant and to cellulose product [6].

The aim of this study is to show that the new microfibrillar collagen hemostat Sangustop^® ^is not inferior to the carrier-bound fibrin sealant Tachosil^® ^with regards to haemostatic efficacy. The efficacy of Tachosil^® ^has been shown in many clinical studies in various indications and thus can be seen as a standard treatment superior to other locally applicable agents [4,5].

## Methods/Design

### Objectives

This study is designed as a prospective, single blinded, randomized, 2-arm trial of two haemostatic products. The primary objective of the study is to show the non-inferiority of Sangustop^® ^(Treatment Arm 1) versus Tachosil^® ^(Treatment Arm 2) in achieving haemostasis after hepatic resection.

#### Primary endpoint

▪ Haemostasis 3 minutes after application of the haemostatic product.

#### Secondary endpoints

▪ Haemostasis 5 minutes after application of the haemostat product.

▪ Haemostasis 10 minutes after application of the haemostat product.

▪ Time to haemostasis

▪ Complications and adverse events intra-operatively during liver resection and during 3 months of follow-up.

### Interventions

#### Surgery

Liver resection (segmental or non-segmental) will be performed according to accepted surgical standards. Surgeons must be board certified and must have a minimum experience of 10 liver resections performed. A standardization of surgical technique will be done inasmuch as there is a restriction in the methods allowed for resection and for primary haemostasis.

The following techniques of liver resection are allowed:

▪ Cavitron Ultrasonic Aspiration (CUSA^®^)

▪ Hydrojet (pressurized jet of water)

▪ Clamp Crushing

▪ Scissors

▪ Stapler transsection

The following techniques of liver resection are not permitted:

▪ Argon beamer (Argon-Plasma Coagulation)

▪ Radiofrequency-assisted devices for parenchymal division (e.g. Habib™-Sealer, TissueLink™)

▪ Ultrasonic dissection (e.g. UltraCision^®^)

The following methods of primary haemostasis are allowed:

▪ Vascular clips

▪ Sutures

All other methods of primary haemostasis (e.g. Argon Laser, bipolar coagulation) are not permitted.

After primary haemostasis has been achieved and with persistent parenchymal bleeding the patient will be randomized to one of two treatment groups. Either Sangustop^® ^or Tachosil^® ^will be applied to the resection area according to the respective instructions for use and the time to haemostasis will be recorded.

#### Follow-up

After surgery, two follow-up examinations will be performed while the patients are still hospitalised. Patients are observed for 3 months for documentation of adverse events. See Table 1 for detailed follow-up.

**Table 1:** Flow-Chart: Overview Clinical Trial Study Activities

Visit	1 Screening	2 Surgery	3 Follow-up 24 hrs after surgery	4 Follow-up 7 days after surgery	5 Follow-up 30 after surgery	6 Follow-up 3 months after surgery
Informed consent	x					

Demographics	x					

Medical history	x					

Medication history	x					

Physical examination	x					

Blood and urine examinations	X**^1, 4^**	X**^1^**	X**^1^**			

Eligibility Criteria	X**^2^**	X**^3^**				

Randomisation		X				

Liver resection (Resection area, weight, histopathology, prim. haemostasis)		X				

Investigational treatment		Sangustop^®^/Tachosil^®^				

Number of Sangustop^® ^Compresses/Tachosil^® ^Sponges used		X				

Time to haemostasis		X				

Required blood transfusions				X		

Central venous pressure		X				

Adverse events incl. complications		X	X	X	X	X

^1^Blood: INR, PTT, Hb, Ht; ^2^1st part; ^3^2nd part, ^4^Blood or urine: pregnancy test.

#### Study materials

Sangustop^® ^(B. Braun Aesculap, Tuttlingen, Germany) is a felt-like haemostatic agent, composed of native absorbable collagen fibrils of bovine origin and containing riboflavin as a colouring agent.

TachoSil^® ^(Nycomed, Linz, Austria) is a ready-to-use fixed combination of a patch sponge (equine collagen) coated with a dry layer of the human coagulation factors fibrinogen and thrombin.

Both investigational products of this clinical trial are approved for clinical use (CE-mark and EU Marketing Authorisation, respectively). They will be used according to respective instructions for use within their usual range of indication.

### Trial population

Clinical trial participants will be recruited in the clinical trial centres consecutively from a population of patients who are planned for an elective liver resection. A detailed overview of the eligibility criteria is given in Table 2. One part of the eligibility criteria are checked preoperatively. A second set of criteria must be checked intra operatively. Patients who do not meet the second part of the eligibility criteria will neither be randomised nor treated with an investigational product. Their trial participation will end by this time. Regular monitoring visits will be performed and the eligibility of all enrolled patients will be checked by source data verification. Resection area will be calculated with the aid of a paper blot of the resection surface.

**Table 2:** Eligibility Criteria

Inclusion Criteria	Exclusion Criteria
▪ Age: >18 years ▪ Patients with an indication for liver resection (segmental or non-segmental) ▪ Willing and able to complete the clinical trial procedures, as described in the protocol ▪ Signed written informed consent to participate in this clinical trial	***Criteria to be checked at screening visit:*** ▪ Presence or sequelae of coagulation disorder, liver cirrhosis, Klatskin tumour ▪ Concurrent participation in another clinical trial with a medical device or medicinal product or with interfering endpoints ▪ Concurrent or previous therapy with systemic pharmacologic agents promoting blood clotting including but not limited to tranexamix acid, activated factor VII, and aprotinine ▪ Known allergy or hypersensitivity to a component of the investigational treatments Sangustop^® ^or Tachosil^®^, to riboflavin or to proteins of bovine origin ▪ Pregnancy or breast feeding ▪ Inability to understand the nature and the extent of the trial and the procedures required
	***Criteria to be checked during surgery:*** ▪ Resection area estimated by operating surgeon <16 cm^2^ ▪ Infected wound area ▪ Persistent major bleeding after primary haemostasis ▪ No bleeding after resection

### Sample size

The sample size was calculated to test for non-inferiority of Treatment Arm 1 (Sangustop^®^) versus Treatment Arm 2 (Tachosil^®^) in terms of haemostasis 3 minutes after application of the haemostatic product. The proportion of patients with a complete haemostasis 3 minutes after application will be assessed. In a previous study [4] the proportion of patients with complete blood clotting after 3 minutes for Tachosil^® ^was estimated to be 73%.

With 60 subjects in each group, the lower limit of the observed one-sided 97,5% confidence interval will be expected to exceed -0,100 with 93% power when the proportion of Treatment Arm 2, π_S_, is 0,730 and the expected proportion of Treatment Arm 1, π_T_, is 0,880; results are based on 100 simulations using the Newcombe-Wilson score method to construct the confidence interval [7].

In case of non-inferiority an additional two group χ^2^-test with a 0,050 two-sided significance level will have 83% power to detect the difference between an Treatment Arm 2 proportion, π_1_, of 0,730 and an Treatment Arm 1 proportion, π_2_, of 0,930 (odds ratio of 4,914) when the sample size in each group is 60.

**Figure 1:**
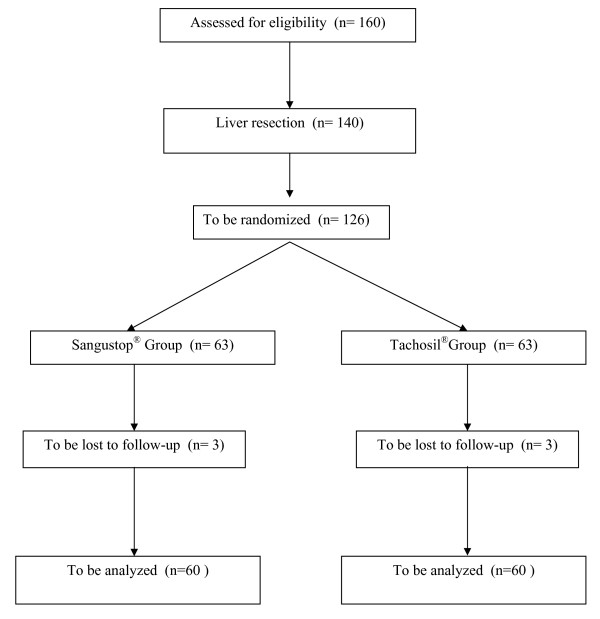
**Flow-Chart acc. to CONSORT**.

Assuming a drop-out rate of 5% a total number of 126 patients needs to be enrolled. Figure 1 shows a flow diagram of the progress through the trial phases.

### Randomisation and blinding

Randomisation is designed in a way that both treatment arms will have the same number of patients, with a randomisation ratio of 1:1 providing equal probability of assignment to each of the two treatment arms. Randomisation is stratified by clinical trial centre and will be performed as block randomization. A separate randomisation list will be created for each clinical trial centre using the Software RandList of the DatInf GmbH, Tübingen, Germany.

Only if a patient fulfils all eligibility criteria he/she will be randomised to an investigational treatment. This will take place during surgery, since part of the exclusion criteria can only be checked during the operation (liver resection). For allocation of the randomisation numbers to eligible trial participants each clinical trial centre will be provided with a set of identical look, opaque and well sealed envelopes. If a patient turns out to be eligible the investigator opens the next envelope in sequence. Inside he will find the randomisation number and the allocated investigational treatment. The sequence of opening the envelopes will be monitored regularly.

This is a single blinded clinical trial. The clinical trial patients will not be informed about their assignment to a study group. The randomization takes place in the operating room immediately before the application of the product. Thus the investigator does not know which product will be used during transection, during primary haemostasis, and when assessing the intra-operative inclusion criteria. However, the appearance of the products precludes a complete blinding of the investigators.

### Statistical analysis

#### Primary Variable

The proportion of patients with haemostasis 3 minutes after application of the haemostat product as well as associated 95% confidence intervals will be reported for each treatment arm. The study null hypothesis is that Tachosil^® ^is more effective than Sangustop^®^. Non-inferiority will be demonstrated if the lower limit of the observed two-sided 95% confidence interval of the observed difference in proportions of Treatment Arm 1 - Treatment Arm 2 does not fall below -0.100. The analysis will be based on the per-protocol population and repeated for the intent-to-treat population. The intent-to-treat population consists of all consenting patients randomized into the study. The safety population comprises all treated patients. The Per-Protocol population excludes all patients who violated inclusion or exclusion criteria of the protocol.

If the 95% confidence interval for the treatment effect not only lies entirely above -0.1 but also above zero then there is evidence of superiority in terms of statistical significance at the 5% level. The difference in proportions between the two treatment arms (Treatment Arm 1 versus Treatment Arm 2) will be tested with a Fisher's exact test to reject the null hypothesis of no difference.

#### Secondary Variables

The proportion of patients with haemostasis 5 minutes and 10 minutes after application will be reported descriptively based on intent-to-treat population as well as the per-protocol population. This will include the proportions, the estimated difference in proportions and the associated 95% confidence intervals. Differences in time to haemostasis will be tested with a log-rank test at the 5% alpha level. Kaplan Meier curves will be displayed, with median estimates and confidence limits provided. The analyses will be based on the intent-to-treat population and repeated for the per-protocol population.

### Trial organization

The trial is initiated and sponsored by B. Braun Aesculap. The sponsors role during study conduct is limited to the project co-ordination. An external Clinical Research Organisation (Centrial GmbH, Tübingen, Germany) is engaged for monitoring, biometry, and data management.

Patients will be recruited by seven German and one Austrian hospitals. Sites are selected according their experience in liver surgery and their willingness to adhere to the study protocol. The participating centres are listed at the end of this paper.

The trial is performed according to the Declaration of Helsinki in its current German version, the national laws for medical devices and for drugs, and the guidelines for Good Clinical Practice (GCP), as applicable. The study was approved by the ethics committee of the Johann Wolfgang Goethe-University, Department of Medicine, Frankfurt, Germay; approval number 197/09.

### Data management and quality assurance

Data is entered in prepared Clinical Report Forms (CRF). Completed CRF pages are checked by the responsible monitor with respect to completeness and plausibility. The data will be transferred into an electronic data processing system by the CRO (CenTrial GmbH). A double data entry will be performed with a double data check. During the recruitment phase the centres will be monitored according to GCP guidelines by qualified monitors from the CRO.

### Ethical aspects

The study was approved by the Ethics Committee of the University of Frankfurt. Secondary approvals will be obtained from all ethics committees responsible for the participating centres. Written informed consent will be obtained from all study subjects before enrolment into the study.

### Current status and duration of the trial

The study protocol has been completed in January 2009. A first investigator meeting was held in April, 2008, where the key points of the study design were discussed and agreed on by all clinical trial centres. Patient recruitment started in January, 2010 and is planned to require 12 months. At the time of submission recruitment is active in six of the eight participating centres.

## Discussion

Postoperative bleeding and biliary leakage are - despite advancement in resection technique - major determinants of morbidity after liver resection, encountered in 4% to 7% of patients [8]. Topical haemostats, glues and sealants are used regularly in liver surgery trying to prevent complications resulting from haemorrhage. The ideal haemostat should be efficient in achieving fast and durable haemostasis and not causing any adverse affects. Additionally, it should be easy to use and cost efficient.

The present study aims to show that the efficacy of a new collagen based haemostyptic agent (Sangustop^®^) is not inferior to another product with a proven efficacy (Tachosil^®^). A non-inferiority study design seems to be appropriate, since there is good evidence from controlled trials that the active control is efficacious. The expected margins of a possible difference are so small - in the range of seconds or few minutes regarding time to haemostasis - that the clinical relevance of a superiority would be at least questionable. In case Sangustop^® ^proves to be no worse than Tachosil^®^, other criteria like incompatibility with the use of human blood components or cost-effectiveness could be taken into consideration. With haemostats potentially being used in all surgical procedures and with the relatively high costs of fibrin sealants and similar products, the cost-effectiveness is of a high importance. The results of this study could influence the choice of haemostatic therapy in a great many cases, especially with extensive parenchymatic bleeding, such as in hepatic resections.

Purified, microfibrillar collagen products have been introduced as haemostats in the 1970ies and have become valuable surgical adjunctives. They induce blood clotting very fast, have a strong adherence to the surface with low tissue reaction and fast resorption [9]. Preclinical data on the new collagen product Sangustop^® ^showed very good haemostatic activity. In a pig liver resection model time to haemostasis was shorter than for Tachosil^® ^or for oxidized cellulose [6].

Some evidence from randomized controlled trials (RCT) exists regarding the use of fibrin sealants on their own or combined with a carrier material [2]. The efficacy of Tachosil^® ^which consists of a collagen fleece carrying the fibrin glue components human fibrinogen and human thrombin has been shown in many clinical studies in various indications and can be seen as a standard treatment superior to other locally applicable agents [4,5]. It was shown in a randomised controlled trial to be superior in obtaining intra-operative haemostasis over argon beamer in liver resection [4,10]. The study by Frilling et al. will serve as the reference for this study design and conduct. Participants, interventions and outcome measures are chosen to be similar or equivalent. Three study centres of the ESSCALIVER - Study (Graz, Heidelberg, München Grosshadern) participated in the reference study, too.

Patients undergoing elective hepatic resection are generally eligible for the study. Liver surgery is to be performed according to accepted surgical standards. For primary surgical haemostasis only sutures and clips are allowed.

The efficacy parameter is "time to haemostasis" which is assessed every minute as presence or absence of haemostasis. Different from the reference study the primary outcome measure is the proportion of patients who achieved haemostasis at 3 minutes. The reference study for Tachosil^® ^as well as the pre-clinical data for Sangustop^® ^suggest that at 3 minutes in the majority of cases (>70%) haemostasis will be achieved already. Thus the interesting "time to haemostasis" is the time between 1 and 3 minutes. However, the instructions for use for Tachosil^® ^require to press the device for at least 3 minutes to the wound surface, preventing a measurement before that time. Therefore, the proportion of patients with haemostasis after 3 minutes was chosen as the appropriate primary outcome. Patient proportions at other time points and the time to haemostasis (Kaplan Meier curves) will be assessed as secondary parameters.

A limitation of the study is that due to the nature of the products a blinding of the observer is not possible. Additionally, the assessment of the time to haemostasis is subjective and prone to bias. However, there is no better method available. This is reflected by the fact that it is the method of choice in comparable studies [2-6,10]."

The margins of non-inferiority are a crucial decision in a non-inferiority trial [11]. The basis for the margin definition in this study were: the reference study, the preclinical data, and expert opinions. Preclinical data suggest that Sangustop^® ^is more efficient in achieving haemostasis than Tachosil^®^. The reference study shows that at 3 minutes in ~70% of patients haemostasis was achieved in contrast to ~50% (difference of ~20%) with the comparator Argon Laser (figures are estimates from the published graphs). In the present study the margins for non-inferiority are set to 10% which firstly is much smaller than the effect of Tachosil^® ^found in the reference study, and secondly was decided by the experts in a study meeting with all participating centres.

Since the comparator is a pharmaceutical product the study has to be conducted under the strict regulations of the national drug law. On the other hand it will additionally assure a high quality study conduct which is a criterion for the acceptance of a non-inferiority study results [11].

Diffuse bleeding from resection surfaces of parenchymatous organs remain a challenge in visceral surgery. Pre-clinical data suggest that Sangustop^® ^might be a very efficient haemostat, however, without any pharmaceutically active plasma components. The present study aims to assess if Sangustop^® ^is non-inferior to the "gold-standard" product Tachosil^®^. In case Sangustop^® ^proves to be as efficient as Tachosil^®^, this will allow surgeons to look at additional criteria like handling characteristics or the cost-effectiveness for the selection of the most appropriate treatment for hemostasis.

## Competing interests

The study and the publication of the study protocol are sponsored by B. Braun Aesculap.

## Authors' contributions

CM, VH, MV, HPK, and WOB participated in the conception and design of the study. GH is responsible for statistical planning and analysis and contributed to the study design. CM and VH drafted the manuscript. All authors have read and approved the manuscript.
